# Quality of life in purely ocular myasthenia in Japan

**DOI:** 10.1186/1471-2377-14-142

**Published:** 2014-07-05

**Authors:** Shigeaki Suzuki, Hiroyuki Murai, Tomihiro Imai, Yuriko Nagane, Masayuki Masuda, Emiko Tsuda, Shingo Konno, Satoru Oji, Shunya Nakane, Masakatsu Motomura, Norihiro Suzuki, Kimiaki Utsugisawa

**Affiliations:** 1 Department of Neurology, Keio University School of Medicine, 35 Shinanomachi, Shinjuku-ku, Tokyo 160-8582, Japan; 2 Department of Neurology, Iizuka Hospital, Fukuoka, Japan; 3 Department of Neurology, Sapporo Medical University, Hokkaido, Japan; 4 Department of Neurology, Hanamaki General Hospital, Iwate, Japan; 5 Department of Neurology, Tokyo Medical University, Tokyo, Japan; 6 Department of Neurology, Toho University Ohashi Medical Center, Tokyo, Japan; 7 Department of Neurology, Saitama Medical Center, Saitama Medical University, Saitama, Japan; 8 Department of Neurology, Nagasaki Kawatana Medical Center, Nagasaki, Japan; 9 Department of Clinical Neuroscience and Neurology, Graduate School of Biomedical Sciences, Nagasaki University, Nagasaki, Japan

**Keywords:** Ocular myasthenia, Ocular-quantitative myasthenia gravis score, Quality-of-life, Therapeutic outcome

## Abstract

**Background:**

Since there has been no conclusive evidence regarding the treatment of ocular myasthenia, treatment guidelines were recently issued by the European Federation of Neurological Societies/European Neurological Society (EFNS/ENS). However, the therapeutic outcomes concerning the quality-of-life (QOL) of patients with ocular myasthenia are not yet fully understood.

**Methods:**

We investigated the therapeutic outcomes of patients with purely ocular myasthenia in a multicenter cross-sectional survey in Japan. To evaluate the severity of ocular symptoms, we used the ocular-quantitative MG (QMG) score advocated by Myasthenia Gravis Foundation of America. We used the Japanese translated version of the MG-QOL15, a self-appraised scoring system.

**Results:**

Of 607 myasthenia gravis (MG) patients with an observation-duration of illness ≥ 2 years, the cases of 123 patients (20%) were limited to ocular muscles (purely ocular myasthenia). During the entire clinical course, 81 patients experienced both ptosis and diplopia, 36 had ptosis alone, and six had diplopia alone. Acetyl-cholinesterase inhibitors and prednisolone were used in 98 and 52 patients, respectively. Treatment improved ocular symptoms, with the mean reduction in ocular-QMG score of 2.3 ± 1.8 points. However, 47 patients (38%) failed to gain minimal manifestation or a better status. Patients with unfavorable outcomes also self-reported severe QOL impairment. Multivariate analyses showed that the pretreatment ocular-QMG score was associated with unfavorable outcomes, but not associated with the patient’s QOL.

**Conclusion:**

A treatment strategy designed in accord with a patient's ocular presentation must be considered in order to improve ocular symptoms and the patient's QOL.

## Background

Ocular myasthenia is a form of myasthenia gravis (MG) that is clinically restricted to extrinsic ocular muscles [1,2]. Clinical signs of ocular myasthenia can be highly variable, ranging from mild unilateral ptosis to complete opthalmoplegia. Ptosis and diplopia may be present, involving various combinations of the levator palpebrae, the two obliques, and the four recti muscles. It is thought that these extraocular muscles have less prominent synaptic folds and/or lower expressions of complement regulators, which makes these muscles vulnerable to autoimmune attacks [1,3]. Almost one-half of MG patients present with ocular symptoms, and 50%–60% progress to the generalized disease, mostly within the first 2 years [4,5]. The percentage of patients with MG who suffer from purely ocular symptoms during the entire course has been reported to be 12%–20% of the whole MG population [1,2,4,5].

Several studies reported that corticosteroid was effective for preventing the progression from ocular myasthenia to generalized MG [6-11]. However, a systematic review concluded that there was no clear evidence supporting corticosteroid use for ocular myasthenia [12]. In addition to generalization from ocular myasthenia, the treatment of ocular symptoms in purely ocular myasthenia has varied, since neurologists had to select treatment regimens based on the particular ocular symptoms of their patients. In this context, the European Federation of Neurological Societies/European Neurological Society (EFNS/ENS) guidelines for the treatment of ocular myasthenia were recently published [13]. To the best of our knowledge, there are no reports of therapeutic outcomes that include the patients’ quality-of-life (QOL) in a large number of patients with purely ocular myasthenia.

The purpose of the present study was to investigate the therapeutic outcomes of purely ocular myasthenia, including QOL measures, in a cross-sectional survey from numerous centers in Japan.

## Methods

Eleven neurological centers participated in the present study as the Japan MG Registry Group. We evaluated patients with established MG who attended these centers between April and July 2012. To avoid potential bias, we enrolled consecutive patients with various stages of illness over a short duration in this multicenter cross-sectional study. All clinical information was collected after the patients gave their written informed consent. All study protocols were approved by the ethics committee of Keio University Hospital, the ethics committee of Hanamaki General Hospital, the ethics committee of Iizuka Hospital, the ethics committee of Sapporo Medical University Hospital, the ethics committee of Saitama Medical Center, the ethics committee of Tokyo Medical University Hospital, the ethics committee of Toho University Medical Center Ohhashi Hospital, the ethics committee of Sendai Medical Center, the ethics committee of Tohoku University Hospital, the ethics committee of Nagasaki University Hospital, and the ethics committee of Nagasaki Kawatana Medical Center.

The diagnosis of MG was based on clinical findings (fluctuating symptoms with easy fatigability and recovery after rest) with amelioration of symptoms after an intravenous administration of acetyl-cholinesterase (AChE) inhibitors, decremental muscle response to a train of low-frequency repetitive nerve stimuli, or the presence of antibodies against skeletal muscle acetylcholine receptor (AChR) [1]. We excluded other disorders which caused ptosis and/or diplopia using various examinations, especially in anti-AChR negative patients. At some of the participating institutions, single-fiber electromyography was used to detect jitter phenomena in the orbicularis oculi muscles.

Clinical information was retrospectively obtained by reviewing the patients’ clinical charts. The patients’ clinical features were evaluated according to the Task Force of the Medical Advisory Board of the Myasthenia Gravis Foundation of America (MGFA) [14]. To evaluate the severity of ocular symptoms, we used the MGFA ocular-quantitative MG (QMG) score, which includes levator function, extraocular muscle function, and the strength of orbicularis oculi among the 13 items of the QMG score [15]. Disease subtypes were classified into early-onset, late-onset, and thymoma-associated MG [16]. Therapeutic outcomes were assessed by the MGFA post-interventional status and the patients’ QOL. We used the Japanese translated version of the MG-QOL15 (MG-QOL15-J) [17], a self-appraised scoring system. It was simple, easy to administer, user-friendly, and quick to assess the impact of a disease.

The spectrum of treatment included AChE inhibitors, corticosteroids, other immunosuppressants, high-dose intravenous methylpredonisolone pulse therapy (mPSL), high doses of immunoglobulin (IVIg), plasmapheresis and extended thymectomy. Among the AChE inhibitors drugs, pyridostigmine bromide (Mestinon®) was usually used. Among oral corticosteroids, prednisolone (PSL) was generally used. The purpose of an additional use of immunosuppressants was to taper the dose of PSL and to reduce the side effects of PSL. Since the use of tacrolimus and cyclosporine was approved by the Japanese Ministry of Welfare in 2000 and 2006, other immunosuppressants including azathioprine and mycophenolate are principally not used in Japan. Plasmapheresis was performed for immunoadsorption using a tryptophan column (TR-350, Asahi Medical, Tokyo). The therapeutic decisions of ocular myasthenia had not been made based on the confirmed protocol, however, the principal treatment methods were similar in all participating institutions in accordance with the guidelines [13].

Comparisons between the favorable and unfavorable outcomes were made using the chi-square test or the Mann–Whitney U-test when appropriate. A multivariate analysis was performed using the Cox proportional hazards regression model to determine independent factors associated with unfavorable outcomes. The statistical analyses were performed using IBM/SPSS software (version 20; Armonk, NY).

## Results

For the investigation of the clinical features of purely ocular myasthenia, MG patients with an observation-duration of illness ≥ 2 years were enrolled. Of 607 MG patients, 123 (20%) patients who showed the worst condition (graded MGFA class 1) were regarded as having purely ocular myasthenia. Their mean age was age 60.9 ± 15.8 years, and the male/female ratio was 54:69. The observational period was 7.8 ± 6.0 years. Forty-one patients had early-onset MG, 68 patients had late-onset MG, and the cases of 14 patients were thymoma-associated. During the entire clinical course, 81 patients (66%) experienced both ptosis and diplopia, 36 (29%) had ptosis alone, and six (5%) had diplopia alone. Although anti-AChR antibodies were detected in 92 patients, no patients had anti-muscle-specific tyrosine kinase antibodies.

The patients’ treatments included AChE inhibitors in 98 patients (80%), oral PSL in 52 (42%), other immunosuppressants in 28 (23%), mPSL in 23 (19%), plasmapheresis in 6 (5%), IVIg in 1 (1%), and thymectomy in 31 (25%). The current dose of pyridostigmine was 101.0 ± 66.7 mg/day. The maximum dose of PSL was 20.4 ± 13.0 mg/day.

**Figure 1:**
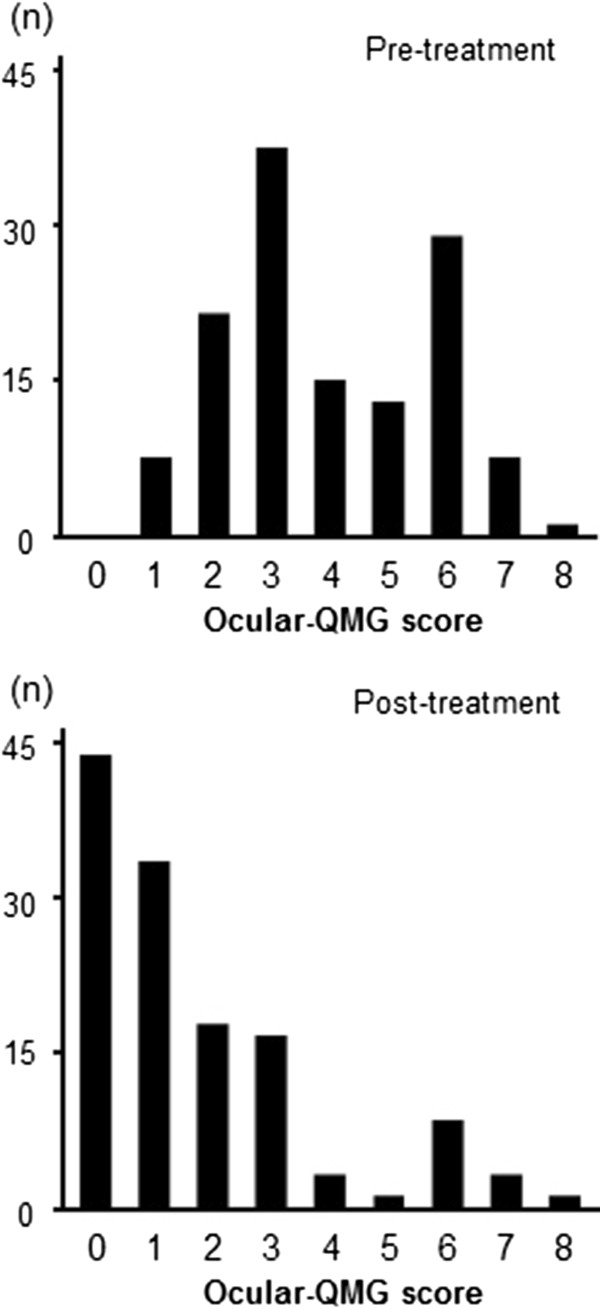
Distribution of pre- and post-treatment ocular-quantitative myasthenia gravis (QMG) scores of the 123 patients with purely ocular myasthenia.

Figure 1 shows the distribution of pre- and post-treatment ocular-QMG scores in all 123 patients with purely ocular myasthenia. The mean reduction in ocular-QMG score was 2.3 ± 1.8 points, and the reductions ranged from 4.0 ± 1.8 points to 1.7 ± 2.0 points. The MGFA post-interventional status was ‘complete stable remission’ in 16 patients (13%), ‘pharmacological remission’ in six (5%), ‘minimal manifestation (MM)’ in 54 (44%), ‘improved’ in 23 (19%), and ‘unchanged’ in 24 (20%). We considered MM or better status (i.e., pharmacological remission or complete stable remission) as a practical treatment goal [17]. Seventy-six (62%) of all patients with ocular myasthenia achieved MM or better status (i.e., a favorable outcome). In contrast, 47 (38%) patients failed to gain MM or better status (i.e., an unfavorable outcome).

We divided the 123 patients with purely ocular myasthenia into two groups: 76 patients with the favorable outcome and 47 patients with the unfavorable outcome. The demographic and clinical features of the two groups are given in Table 1. There were no significant between group differences in gender, age, observational period, ocular symptoms, disease subtype, or seropositivity of anti-AChR antibodies. In addition, there were no significant differences in the treatment profiles between the two groups. The current dose of pyridostigmine was significantly higher in the patients with unfavorable outcomes than those with favorable outcomes (132.0 ± 54.0 mg vs. 79.7 ± 66.6 mg, p < 0.0001).

**Table 1:** The favorable and unfavorable outcome groups among 123 patients with purely ocular myasthenia

	**Favorable outcome**	**Unfavorable outcome**	
	**(n = 76)**	**(n = 47)**	**p-value**
Female	42 (55%)	27 (57%)	0.81
Age, yr	61.1 ± 16.1	60.4 ± 15.4	0.81
Observational period, yr	7.2 ± 4.6	8.7 ± 7.7	0.22
Subtype			
Early-onset	23 (30%)	18 (38%)	0.33
Late-onset	42 (55%)	26 (55%)	1.00
Thymoma-associated	11 (14%)	3 (6%)	0.17
Symptoms			
Ptosis and diplopia	49 (64%)	32 (68%)	0.68
Ptosis alone	22 (29%)	14 (30%)	0.92
Diplopia alone	5 (7%)	1 (2%)	0.26
Anti-acetylcholine receptor positive	60 (79%)	32 (68%)	0.18
Treatment			
Acetyl-cholinesterase inhibitors	58 (86%)	41 (78%)	0.14
Pyridostigmine (mg/day)	79.7 ± 66.6	132.0 ± 54.0	<0.0001
Oral prednisolone	27 (36%)	25 (53%)	0.053
Maximum dose (mg/day)	19.4 ± 10.4	21.5 ± 15.5	0.58
Immunosuppressants	14 (18%)	14 (30%)	0.14
mPSL	11 (14%)	12 (26%)	0.13
Plasmapheresis	2 (3%)	4 (9%)	0.14
Immunoglobulin	1 (1%)	0 (0%)	0.43
Thymectomy	20 (26%)	11 (23%)	0.71
Ocular-QMG score			
Pre-treatment	3.4 ± 1.7	4.5 ± 1.7	0.006
Post-treatment	0.6 ± 0.8	3.4 ± 2.1	<0.0001
MG-QOL15-J score	5.7 ± 8.5	15.7 ± 12.7	<0.0001

mPSL, intravenous methylpredonisolone pulse therapy.

QMG, quantitative MG score; MGFA, Myasthenia Gravis Foundation of America.

**Figure 2:**
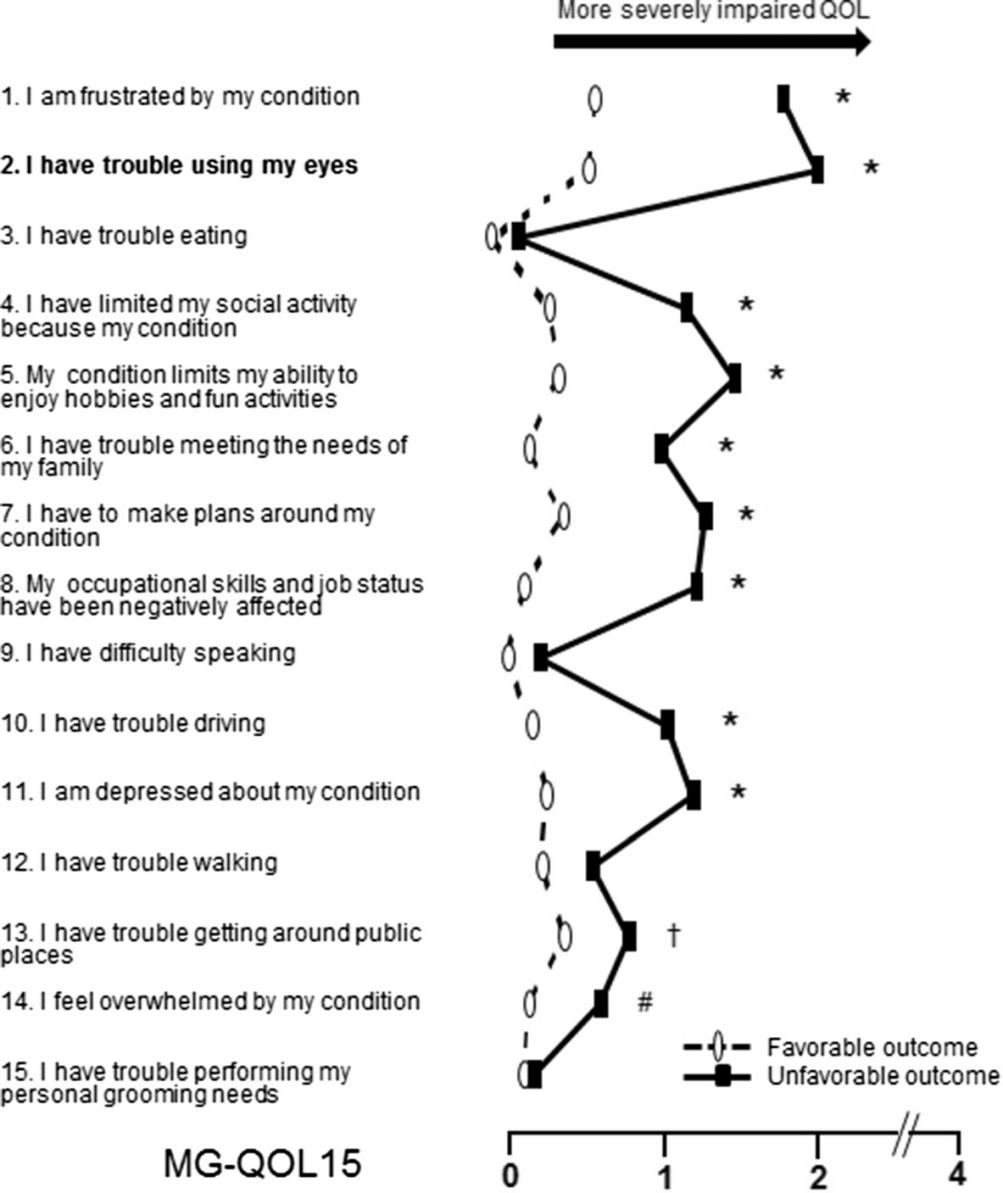
**Scores on the 15 items of MG-QOL15-J. Statistical analyses were performed between the unfavorable (n = 47) and favorable (n = 76) outcome groups.** †p < 0.05, #p < 0.01, and *p < 0.001.

The pretreatment ocular-QMG scores were significantly higher in the patients with unfavorable outcomes compared to those with favorable outcomes (4.5 ± 1.7 vs. 3.4 ± 1.7 points, p = 0.006). We conducted logistic analyses to identify the clinical factors associated with the unfavorable outcome. We examined the association between the unfavorable outcome and the clinical factors of gender, age, observational period, ocular symptoms, disease subtype, seropositivity of anti-AChR antibodies, and treatment profiles. The multivariate logistic regression analyses revealed that the pretreatment ocular-QMG score was the only factor associated with the unfavorable outcome (odds ratio = 1.382, 95% confidence interval, 1.084–1.761, p = 0.009).Lastly, we evaluated the self-perceived QOL of patients with purely ocular myasthenia by the MG-QOL15-J. Forty-seven patients with an unfavorable outcome showed significantly higher scores on the total MG-QOL15-J (more severely impaired QOL) compared to the 87 patients with the favorable outcome (15.7 ± 12.7 vs. 5.7 ± 8.5 points, p < 0.0001). The comparison of the 15 items of the MG-QOL-15 is shown in Figure 2. Patients with the unfavorable outcome scored themselves as "severe" for 11 of 15 items of the QOL survey, more than those with the favorable outcome. Although we also examined the association between the QOL and the clinical factors of ocular myasthenia and treatment profiles using the multivariate logistic analyses, we could not find the significant factor associated the self-perceived QOL.

## Discussion

The present multicenter cross-sectional survey in Japan was comprised of the treatment profiles and therapeutic outcomes of 123 patients with purely ocular myasthenia. To investigate the clinical features of purely ocular myasthenia, we enrolled MG patients with durations of symptoms ≥2 years. The main results can be summarized as follows: (i) the frequency of purely ocular myasthenia was 20% in all MG patients; (ii) treatment improved the ocular symptoms with the mean reduction in ocular-QMG score of 2.3 ± 1.8 points; (iii) 38% of the patients failed to gain MM or a better status; (iv) the unfavorable outcome was also demonstrated by severe QOL impairment; and (v) the multivariate analyses showed that the pretreatment ocular-QMG score was associated with unfavorable outcomes, but not associated with patient’s QOL.

The present study has two limitations. First, the cross-sectional study did not allow us to draw any conclusions about the treatment selection of ocular myasthenia. Second, we obtained no data regarding the association between drug therapy and generalization from ocular myasthenia. An evidence-based review made no conclusion regarding whether it was appropriate to initiate therapy with AChE inhibitors or with corticosteroids for patients with ocular myasthenia [12]. However, the EFNS/ENS guidelines recommend that the treatment of ocular myasthenia should initially be started with pyridostigmine [13]. We agree with the principal concept of the EFNS/ENS guidelines’ recommendation and initially treated by AChE inhibitors in most patients with ocular myasthenia. However, we should recognize that AChE inhibitors alone usually will not solve the ocular myasthenia. We emphasize that the indication for immunosuppressive treatment should be carefully considered based on the severity of ocular symptoms in order to improve the patient’s QOL.

The ocular symptoms can impair an individual’s vision enough to interfere with work and daily life, but the QOL of patients with purely ocular myasthenia had not been elucidated prior to the present study. The MG-QOL15-J, useful for identifying satisfaction or dissatisfaction with the manifestations of MG among patients receiving treatment, can capture various aspects of QOL impairment [17]. We found a tight association between the therapeutic outcome and QOL impairment in purely ocular myasthenia. The MG-QOL15-J demonstrated a marked gap (a total of 10 points) between the favorable and unfavorable outcomes. It is likely that the difference may be much greater than physicians’ global impressions.

Apart from the generalization from ocular myasthenia, we must consider the therapeutic strategy for purely ocular myasthenia patients with the unfavorable outcome. The treatment should minimize ocular symptoms with minimal side effects and must be tailored to the individual, based on his or her particular ocular symptoms [12,13,18]. In patients with severe opthalmoplegia, therapy with AChE inhibitors alone may result in irreversible eye movement. We think that immunotherapy can be started when an ocular myasthenia patient has a high ocular-QMG score. In fact, 42% of our patients received PSL with the maximum dose of 20.4 mg/day. Since corticosteroid treatment did not result in the satisfactory control of ocular symptoms, we used steroid-sparing treatment with tacrolimus or cyclosporine in 28% of all patients.

In regard to the other treatments, IVIg and plasmapheresis were performed in some patients. However, we also think that these treatments are not indicated for purely ocular myasthenia [13]. In contrast, we consider that mPSL is a potentially effective treatment for ocular myasthenia. It was reported that repeated mPSL in addition to oral PSL was effective for ocular symptoms without initial worsening [19]. In fact, our present findings revealed that mPSL was administered in 19% of all of the patients with purely ocular myasthenia, and 91% of them experienced effective responses. Thus, we think mPSL is the good choice of patients with the unfavorable condition and effective for improving the patient’s QOL. Extended thymectomy was performed in 17 nonthymomatous patients, only when drug therapy had failed. Among the 14 patients with thymoma-associated ocular MG, 11 had a favorable outcome. However, there was no definite data regarding whether thymectomy was contributed to the favorable outcome.

We believe that the treatment of sole ptosis may necessitate a particular approach. We found that the proportion of patients with ptosis alone during the entire course among all of the MG patients was 6% (36/607). It is likely that corticosteroids are less effective for ptosis than for generalized symptoms of MG [18]. Ptosis can be corrected by the placement of “crutches” in the patient’s spectacles, and ptosis tape elevates the eyelid droop [18]. We also use topical naphazoline for treating myasthenic ptosis. Naphazoline, a primary α2-agonist, selectively increases the tone of Muller muscles without mydriasis and successfully reduces myasthenic ptosis [20]. Although rapid effects of topical naphazoline without significant adverse effects were observed in 70% of the MG patients in a previous study, their responses were variable and temporary [20]. In cases of long-standing and irreversible ptosis, surgery for ptosis may be considered [21].

## Conclusion

A treatment strategy designed in accord with a patient's ocular presentation must be considered to improve the ocular symptoms and the patient's QOL.

## Competing interests

The authors declare that they have no conflict of interests.

## Authors’ contributions

SS had full access to all of the data in the study and takes responsibility for the integrity of the data and the accuracy of the data analysis. HM and TI participated in the design and performed the statistical analyses. YN participated the management of the multicenter cross-sectional survey. MMa, ET, SK, SO, SN, and MMo contributed in acquisition of data. NS and KU contributed in obtaining funding critical revision of the manuscript and study supervision. All authors read and approved the final manuscript.

## Pre-publication history

The pre-publication history for this paper can be accessed here:

http://www.biomedcentral.com/1471-2377/14/142/prepub
